# Complexity of case mix in a regional allergy service

**DOI:** 10.1186/1756-0500-5-103

**Published:** 2012-02-17

**Authors:** Edward R Kaminski, Claire A Bethune, Ray B Jones

**Affiliations:** 1 Department of Clinical Immunology & Allergy, Level 07, Derriford Hospital, Plymouth, PL6 8DH, UK; 2 Faculty of Health, University of Plymouth, 3 Portland Villas, Plymouth, PL48AA, UK

**Keywords:** Allergy, Case-mix, Referrals, Audit

## Abstract

**Background:**

Currently in the United Kingdom (UK), there is a mismatch between limited financial resources and the large proportion of patients with suspected allergies actually being referred to specialist allergy clinics. To better understand the case mix of patients being referred, we audited referrals to a regional allergy service over an 8 year period.

The main source of data was consultant letters to General Practitioners (GP) summarising the diagnosis of patients, archived from January 2002 to September 2009. Letters were reviewed, extracting the clinic date, doctor seen, gender, date of birth, postcode, GP, and diagnoses. Diagnoses were classified into seven groups and illustrative cases for each group noted.

**Findings:**

Data from 2,028 new referrals with suspected allergy were analysed. The largest group of patients (43%) were diagnosed with a type I hypersensitivity. The other diagnostic groups were chronic idiopathic (spontaneous) urticaria (35%), suspected type I hypersensitivity but no allergen identified (8%), idiopathic (spontaneous) angioedema (8%), physical urticaria (2.5%), non-allergic symptoms (1.6%), type IV hypersensitivity (0.8%) and ACE inhibitor sensitivity (0.5%). Two thirds of patients seen were female with a higher percentage of female patients in the non type-I hypersensitivity group (71%) than the type 1 hypersensitivity (66%) (χ^2 ^= 5.1, 1df, *p = 0.024*). The type 1 hypersensitivity patients were younger than other patients (38 Vs 46 years, t = -10.8, *p < 0.001*)

**Conclusions:**

This study highlights the complexity of specialist allergy practice and the large proportion of patients referred with non-type I hypersensitivities, chronic idiopathic (spontaneous) urticaria being by far the largest group. Such information is critical to inform commissioning decisions, define referral pathways and in primary care education.

## Findings

### Background

Currently in the UK, there is a mismatch between limited financial resources and the large proportion of patients with suspect allergies actually being referred or potentially being referred to secondary care. We believe that there is room for more selectivity across the whole range of patient groups with patients with symptoms of lesser severity or complexity being seen in a primary care setting. However, in order to achieve this we need to (i) better understand the case mix of patients being referred, and (ii) support general practitioners (GPs) in the management of patients with allergy and related conditions. This study addresses the first of these aims.

Allergy is becoming an increasing problem both worldwide and in the UK. This is due to an increased incidence, with approximately one third of the UK population developing allergy at some time in their lives, together with increased severity and complexity [1,2]. In the UK there is a deficit of allergy services, discussed in a number of high profile reports from the Royal College of Physicians [1], Clinical Immunologists [3], House of Lords [4], and the Government [5,6]. Allergies are exaggerated immune responses (hypersensitivity) to ubiquitous substances and are divided into four categories, termed type I, II, III and IV hypersensitivity reactions by the Gell & Coombs classification [7]. Type I hypersensitivities account for the majority of cases of allergy and are triggered by IgE mediated reactions involving release of histamine and leukotrienes [7].

Although the above reports highlight the groups of patients that should be seen in specialist allergy clinics there is a scarcity of information on the actual patients currently referred to, and the diagnoses made in, such clinics. Such information is critical to inform commissioning decisions, define referral pathways and in primary care education. There are few estimates of referred and confirmed allergy derived from clinic based studies. Asero et al [8] reported the types of allergy found in 25601 patients attending 17 allergy clinics throughout Italy, and Joral et al [9] reported the numbers of patients with food allergies in 3034 patients referred to two hospitals in the Basque country. One study has also looked at referrals to a primary-care based allergy clinic demonstrating that a primary care allergy service provided by appropriately trained personnel can cater adequately for the majority of primary care referrals [10] but these are limited in the UK.

The Peninsula Allergy Service, based in Derriford Hospital, Plymouth, serves all of Devon and Cornwall in South West England. We recently reported on patients referred to the Peninsula Allergy Service who had been diagnosed with an allergy (type 1 hypersensitivity) in the 11-year period September 1998 to September 2009 [11]. From data extracted from 2788 referrals, 961 patients were diagnosed with an allergy. The types of allergy, patient characteristics and maps showing rates by postcode were reported. That study showed marked geographical differences in allergy referrals which are likely to reflect a combination of environmental factors and GP referral patterns. However, type 1 hypersensitivity only represents a proportion of referrals to UK allergy services. Given the scarcity of allergy services we need to know more about the total pattern of referral including non-allergy cases.

In this study, in order to understand better the case mix of patients seen in secondary care to inform decisions about the use of secondary care resources, we have reviewed the case-mix of referrals to a regional allergy clinic in South West England. We present typical cases that illustrate the meaning of the group headings used.

### Methods

Ethics approval was not required as this was an audit project using de-identified patient data.

Data Collection: The main source of data comprised letters from consultants to GPs summarising the outpatient consultation. The patients with suspected allergy had been referred to the Peninsula Allergy Service from GPs in Devon and Cornwall. We did not have complete details for the period 1998 to the end of 2001 so letters archived from January 2002 to September 2009 were reviewed, extracting the clinic date, doctor seen, gender, date of birth, postcode, GP, and diagnosis.

Data Analysis: Based on the diagnoses a classification system was devised and each case allocated to one of seven categories. We report frequency distributions together with illustrative case reports, chosen as being typical of each category of patient. We used χ^2 ^tests to explore differences by age, gender, and postcode area of residence, with significance set at *p < 0.05*.

### Results

After removal of duplicates, data from 2028 new referrals with suspected allergy were analysed and the final diagnoses summarised in Table 1. Typical presentations of each of the seven categories are described below. Two thirds of patients seen were female with a higher percentage of female patients in the non type-I hypersensitivity group (71%) than the type 1 hypersensitivity (66%) (χ^2 ^= 5.1, 1df, *p = 0.024*). The type 1 hypersensitivity patients were younger than other patients (38 Vs 46, t = -10.8, *p < 0.001*). There was a slight difference by postcode area in the number of Type 1 allergy Vs non allergy (Table 2), with more type 1 allergy referrals from Exeter and Truro (further away from the hospital) (χ^2 ^= 9.5; 3df; *p = 0.02*).

**Table 1:** Diagnostic classification of 2028 referrals to the Peninsula Allergy Service over 8 years

Diagnostic classification	N	%	Mean Age (SD)	% Female
1. Type I hypersensitivity	866	42.7	38 (15.4)	66%
2. Chronic idiopathic (spontaneous) urticaria and/or angioedema	882	43.5	46 (17.1)	71%
3. ?Type I hypersensitivity (no trigger found)	171	8.4	47 (16.2)	71%
4. Physical/viral-induced urticaria	51	2.5	38 (16.2)	69%
5. Non-allergic symptoms	32	1.6	47 (15.1)	63%
6. Type IV hypersensitivity	16	0.8	39 (15.2)	100%
7. ACE inhibitor sensitivity	10	0.5	66 (10.9)	70%
**TOTAL**	2028	100%	41 (17.6)	68%

**Table 2:** Number of patients referred over 8 years, comparing type 1 allergy with non-allergy cases within postcode areas

Postcode area	Type 1 allergy	Non-allergy	Total
Exeter	128 (49%)	132 (51%)	260
Plymouth	487 (41%)	694 (59%)	1181
Torquay	181 (41%)	266 (59%)	447
Truro	70 (50%)	70 (50%)	140
TOTAL	866 (43%)	1162 (57%)	2028

Below we outline the diagnoses representative of the referrals received and analysed.

1. Type 1 hypersensitivity: 43% of patients were diagnosed with a type I hypersensitivity making up the largest group. The sub classification and more detailed description of this group have been presented elsewhere [10]. The predominant presenting symptoms in this group of patients were urticaria, angioedema, anaphylaxis, rhinitis, conjunctivitis and wheezing. These patients have an IgE mediated hypersensitivity which, in the majority, can be tested for and confirmed by skin prick testing or specific IgE testing.

*A 23 year old male presented with suspected allergy. He gave a six week history of three episodes of mouth tingling, facial swelling and a nettle-like rash on his trunk, each becoming progressively more severe. Each episode occurred within 5 to 10 minutes of eating peanuts or food containing peanuts. Good response to antihistamines. Skin prick testing to peanuts was positive, confirming the diagnosis of a type I hypersensitivity to peanuts. The patient was advised to avoid peanuts, given a management plan for dealing with future reactions and since then has had no further reactions*.

2. Chronic idiopathic (spontaneous) urticaria and/or angioedema: An equally large group (43%) of patients were those with chronic idiopathic (spontaneous) urticaria and/or angioedema. These patients suffer from chronic recurrent urticaria which can sometimes be associated with angioedema, particularly affecting the face. The first is a good example of a case of an idiopathic reaction consisting predominantly of urticaria although there was also a component of angioedema. However angioedema may present without urticaria as in the second example.

*A 37 year old female presented with suspected allergy. She gave a three month history of episodes of a nettle like-rash and hives affecting her trunk and sometimes associated with facial swelling. The attacks occurred on average every 2-3 days, each lasting approximately 24 hours, generally commencing in the evening or during the night. There was no temporal association with food or drugs. For the past year the patient had had difficulties with her partner and they had split up four months ago. Initially there had been a good response to standard dose antihistamines but more recently the control became sub-optimal. On the basis of the history, a diagnosis of chronic idiopathic urticaria was made and the patient commenced on regular high dose non-sedating antihistamines. This successfully controlled the urticaria until it spontaneously resolved three months later*.

*A 41 year old male presented with suspected allergy. He gave a six months history of five episodes of lip and tongue swelling not associated with a rash. He found the tongue swelling frightening although it had never affected his breathing. The swelling generally occurred during the night and there was no temporal association with food or drugs. The patient was not taking any regular medications and there was no relevant family history. Six months previously he had been made redundant and was struggling to find a new job. Antihistamines helped to a degree but took up to two hours to resolve the swelling. Complement C3 and C4 studies were normal excluding C1 esterase inhibitor deficiency. On the basis of the history and the normal complement studies, a diagnosis of idiopathic angioedema was made. The patient was commenced on regular high dose non-sedating antihistamines which successfully controlled the angioedema until it spontaneously resolved three months later*.

3. ?Type I hypersensitivity (no trigger found): 8.4% of patients had sporadic urticaria and/or angioedema suggestive of a type I hypersensitivity i.e. a temporal association with meals or other triggers but for which no clue could be identified from the history or indeed by allergy testing. These could be either a type I hypersensitivity or could be idiopathic.

*A 23 year old female presented with suspected allergy. She gave a one year history of approximately 10 episodes of mild lip swelling and a nettle-like rash on her neck and upper torso. These generally occurred in the evening within an hour of her evening meal but no suspected food trigger could be identified. There was a good response to antihistamines. A diagnosis of urticaria/angioedema of unknown cause was made and the patient advised to keep a careful food diary and advice given regarding the management of severe episodes*.

4. Physical/viral-induced urticarial: 2.5% of patients had symptoms triggered by physical factors, such as heat, cold, exercise, pressure, water, exercise etc or viral infections

*A 36 year old female presented with suspected allergy. She gave a four year history of recurrent episodes of a nettle-like rash and wheals affecting exposed areas of her skin. There was no temporal association with food or drugs and the only identifiable trigger was cold, particularly cold air. There was a poor response to antihistamines. A diagnosis of cold urticaria was made and the patient advised to take high dose prophylactic non-sedating antihistamines when at risk of exposure to cold and to try to avoid undue exposure to cold. This resulted in a partial improvement in her symptoms*.

5. Non-allergic symptoms: 1.6% of patients had symptoms consistent with an alternative diagnosis where an allergy (type I or other) had definitively been excluded.

*An eighteen year old female presented with suspected allergy. She gave a 9 month history of episodes of abdominal pain and bloating but no association with urticaria or angioedema. She attributed these episodes to an allergy to wheat as the attacks occurred after eating wheat containing foods, generally an hour or two later. Antihistamines had no effect but eliminating wheat from the diet did seem to help. Skin prick testing to wheat was negative excluding a type I hypersensitivity to wheat and a blood test for coeliac serology (after 8 weeks on a normal wheat-containing diet) was negative excluding coeliac disease. On the basis of the history and the negative allergy and coeliac tests, a diagnosis of wheat intolerance was made. The patient was advised to avoid wheat containing products but encouraged to try to re-introduce wheat into her diet every 3-6 months. One year later her symptoms resolved*.

6. Type IV hypersensitivity: 0.8% of patients had a diagnosed type IV hypersensitivity. These patients generally had contact dermatitis to latex, metals or topical drugs.

*A 28 year old female presented with suspected allergy. She gave a two year history of a raised red rash on her ear lobes which would eventually crack and last for several days. There were no other rashes or swellings. The only suspect trigger was her nickel ear rings. The patient was referred to Dermatology where contact dermatitis was diagnosed and patch testing confirmed a type IV hypersensitivity to nickel. The patient was advised to avoid nickel and since then has had no further problems*.

7. Angiotensin converting enzyme (ACE) inhibitor sensitivity: 0.5% of patients were diagnosed with angioedema secondary to an angiotensin converting enzyme (ACE) inhibitor. These patients develop sporadic angioedema of the face while on ACE inhibitors.

*A fifty seven year old male presented with suspected allergy. He gave a nine month history of three episodes of lip and tongue swelling which were not associated with any other swelling or rashes. The attacks always occurred during the night and there was no temporal association with food or drugs. The patient also suffered from hypertension for which he had been taking lisinopril for two years. The lisinopril was stopped, the patient commenced on alternative anti-hypertensive medication and the angioedema resolved, consistent with a diagnosis of ACE inhibitor sensitivity. The patient was advised to avoid ACE inhibitors in future*.

### Discussion

In this study, we have audited referrals to a regional allergy clinic to understand better the case mix of patients seen. It might be thought that a regional allergy service would spend most of its time and resources treating patients with allergy but this audit shows that of patients seen in the clinic only 43% were positively diagnosed as suffering from a type I hypersensitivity. This first group of patients with a diagnosed type I hypersensitivity were discussed in detail as part of a larger series in a previous study [11].

Making an accurate diagnosis is the major clinical intervention that can be made in such patients [12]. However 8.4% of patients seen in the clinic had symptoms suggestive of a type I hypersensitivity (eg reactions with a temporal association with food or other triggers) but for which no evidence of type I hypersensitivity could be elicited from the history or proven by allergy testing. Some of these patients probably did have a type I hypersensitivity to an as yet unidentified allergen but others may have had idiopathic reactions with a coincidental temporal association with food or other triggers. Some of this group had severe symptoms including anaphylaxis.

It is appropriate for patients with symptoms at the more severe end of the spectrum and those where the symptoms are complex or where making a diagnosis requires specialist services (eg challenge testing) to be seen in a regional allergy service. However with adequate GP training and support some of the cases with less severe symptoms could be appropriately managed in primary care.

There were as many patients with chronic idiopathic (spontaneous) urticaria and/or angioedema referred as there were with type 1 hypersensitivity. By definition these patients do not have an underlying allergic trigger [13,14]. Although in a small minority, an underlying infectious or inflammatory focus is found and in others an autoimmune process is the cause of the urticaria/angioedema, in the majority no medical cause can be identified. We have recently demonstrated an association between stressful life events (eg. bereavement, divorce, etc.), posttraumatic stress and poor coping strategies in a large proportion of these patients [15,16]. The difficulty with this group of patients is that both the patient and the GP often incorrectly attribute the cause of the urticaria or angioedema to an allergy, highlighting the complexity of allergy practice.

The fourth group of patients referred were those in whom physical factors, such as heat, cold, exercise, pressure, aquagenic, etc or viral infections triggered the symptoms of urticaria. The diagnosis of this condition is made on the basis of the history and reproducing the physical stimulus on the skin if necessary [17].

The fifth group of patients had symptoms that did not include urticaria, angioedema, rhinitis, conjunctivitis and wheezing but which the patient attributed to an allergy. The public perception of allergy is often not in accord with medical knowledge and results in patients putting pressure on general practitioners to refer to a specialist to 'sort out my allergy'. Although in our study this group of patients was relatively small, in reality it was much bigger as many such referrals were turned away (with a letter of advice) before being accepted in the clinic. The predominant symptoms were gastrointestinal such as abdominal colic and bloating and there was a temporal association between eating the food and the onset of symptoms although this often could be more than one hour. In some cases, a diagnosis of food intolerance was made. Many patients also suffer from irritable bowel syndrome for which there is no test, the diagnosis is made on the basis of a good history and exclusion of other causes [18].

The sixth group of patients suffered from a type IV hypersensitivity to metals, latex or topical drugs especially in creams. This type of hypersensitivity generally triggers a contact dermatitis and not urticaria or angioedema. The diagnosis is made by patch testing, generally performed in Dermatology clinics [19].

The final group of patients in this study comprised those with ACE inhibitor sensitivity. These patients develop sporadic angioedema, particularly affecting the lips and tongue, which often occurs during the night. It is sometimes difficult to diagnosis in view of the fact that the patients have been taking the medication every day, often for several years. There is no diagnostic test for this condition and the diagnosis is made by stopping the ACE inhibitor [20].

Although patients with C1 esterase inhibitor deficiency (hereditary and acquired angioedema) are included in the differential diagnosis of angioedema they were not included in this particular audit as in our clinical service they are classified as a primary immune deficiency.

In summary this audit has clearly shown the burden on the local NHS allergy services of these two major groups of patients, namely those with a type I hypersensitivity and those with chronic idiopathic (spontaneous) urticaria and/or angioedema. It has also highlighted the complexity of cases where the main presentation is urticaria and/or angioedema and the difficulties faced by primary care practitioners in managing these patients. Currently, both locally and nationally in the UK, there is a mismatch between limited financial resources and the large proportion of patients with suspect allergies actually being referred or potentially being referred to secondary care. We believe that, with adequate support from secondary care, there is scope for more patients with symptoms of lesser severity or complexity across the whole range of patient groups being seen in a primary care setting. Naturally patients with severe or complex allergies need to be seen in a specialist allergy clinic. However, in order to achieve this there is a real need for primary care allergy education and support. In the Devon and Cornwall peninsula, over the past few years, much effort has gone into educating GPs in allergy. Regular GP allergy education days have taken place on a yearly basis for the past four years, with attendances often exceeding 100 GPs. In addition a three-day Practical Clinical Allergy course organised by the Peninsula College of Medicine and Dentistry has now been running successfully on a twice yearly basis for the past year.

Although this audit adds to our understanding of the complexity of our allergy service, the study has limitations. We present one case study of referrals up to and including 2009. The case-mix in other regional clinics may not show the same pattern of referral. Also, interventions in the last 2-3 years may have already changed the case-mix of referrals.

### Conclusions

We aimed to understand better the case mix of patients seen in specialist allergy clinics in the UK via an audit of referrals over 8 years to one regional clinic. A complex mix of cases was referred including a large proportion of patients with non-type I hypersensitivities, of which chronic idiopathic (spontaneous) urticaria was the largest group. This information about case-mix is essential to inform commissioning decisions, define referral pathways, and to develop continuing professional development of general practitioners. Further work is needed to determine whether a formal demand management approach would limit referrals to only those that cannot be managed in primary care.

## Competing interests

The authors declare that they have no competing interests.

## Authors' contributions

ERK and RBJ had the idea for the study. RBJ managed the project, carried out initial data cleaning and analysis. ERK and CAB were responsible for the patients in the study and made the clinical diagnoses. ERK, RBJ and CAB all contributed to the writing of the paper. All authors read and approved the final manuscript.
